# Application of real-time quaking-induced conversion in Creutzfeldt–Jakob disease surveillance

**DOI:** 10.1007/s00415-022-11549-2

**Published:** 2023-01-10

**Authors:** Peter Hermann, Matthias Schmitz, Maria Cramm, Stefan Goebel, Timothy Bunck, Julia Schütte-Schmidt, Walter Schulz-Schaeffer, Christine Stadelmann, Jakob Matschke, Markus Glatzel, Inga Zerr

**Affiliations:** 1 Department of Neurology, National Reference Center for CJD Surveillance, University Medical Center Göttingen, Robert-Koch Street 40, 37075 Goettingen, Germany; 2 Deutsches Zentrum für Neurodegenerative Erkrankungen e.V. (DZNE), Göttingen, Germany; 3 Institute of Neuropathology, Medical Faculty of the Saarland University, Homburg, Germany; 4 Institute of Neuropathology, University Medical Center Göttingen, Göttingen, Germany; 5 Institute of Neuropathology, University Medical Center Hamburg-Eppendorf, Hamburg, Germany

**Keywords:** Creutzfeldt–Jakob disease, Prion, Diagnosis, RT-QuIC, Epidemiology

## Abstract

**Background:**

Evaluation of the application of CSF real-time quaking-induced conversion in Creutzfeldt–Jakob disease surveillance to investigate test accuracy, influencing factors, and associations with disease incidence.

**Methods:**

In a prospective surveillance study, CSF real-time quaking-induced conversion was performed in patients with clinical suspicion of prion disease (2014–2022). Clinically or histochemically characterized patients with sporadic Creutzfeldt–Jakob disease (*n* = 888) and patients with final diagnosis of non-prion disease (*n* = 371) were included for accuracy and association studies.

**Results:**

The overall test sensitivity for sporadic Creutzfeldt–Jakob disease was 90% and the specificity 99%. Lower sensitivity was associated with early disease stage (*p* = 0.029) and longer survival (*p* < 0.001). The frequency of false positives was significantly higher in patients with inflammatory CNS diseases (3.7%) than in other diagnoses (0.4%, *p* = 0.027). The incidence increased from 1.7 per million person-years (2006–2017) to 2.0 after the test was added to diagnostic the criteria (2018–2021).

**Conclusion:**

We validated high diagnostic accuracy of CSF real-time quaking-induced conversion but identified inflammatory brain disease as a potential source of (rare) false-positive results, indicating thorough consideration of this condition in the differential diagnosis of Creutzfeldt–Jakob disease. The surveillance improved after amendment of the diagnostic criteria, whereas the incidence showed no suggestive alterations during the COVID-19 pandemic.

**Supplementary Information:**

The online version contains supplementary material available at 10.1007/s00415-022-11549-2.

## Introduction

Human prion diseases are a group of rapidly progressive neurodegenerative disorders that are caused by a conformational change of the physiological Prion protein (PrPC) into its pathogenic isoform Prion protein scrapie (PrP^Sc^). In prion diseases, PrP^Sc^ spreads throughout the central nervous system, forms amyloid aggregations, and ultimately leads to an inevitable spongiform degeneration of the brain. PrP^Sc^ has characteristics of an infectious agent and prion diseases are potentially transmissible [1, 2].

Sporadic Creutzfeldt–Jakob disease (sCJD) is the most frequent human prion disease, followed by hereditary forms that may clinically present as familial CJD (fCJD), Fatal Familial Insomnia (FFI), or Gerstmann Sträussler–Scheinker syndrome (GSS). Acquired prion diseases (iatrogenic CJD and variant CJD) are rare but still a matter of public health [3]. The incidence of sCJD is 1.5–2.0 per million person-years and the median survival time 5–6 months [4, 5]. Distinct clinical and neuropathological phenotypes of sCJD are associated with an interplay of the Methionine (M)/ Valine (V) polymorphism at Codon 129 PRNP and the PrP^Sc^ glycotype (1 and 2) [6], resulting in six major subtypes of sporadic prion disease: MM/MV1, MM/MV2C (cortical), VV2, MV2K (with “kuru-plaques”), VV1, and MM2T (sporadic Fatal Insomnia). These subtypes are also associated with distinct characteristics regarding fluid biomarkers and MRI patterns [7, 8]. Although a definite diagnosis of sCJD is made by neuropathological investigation, clinical criteria have been established decades ago to diagnose a “probable” sCJD [9]. Subsequently, fluid markers (proteins 14-3-3) [10] and MRI [11] have been added as biomarker criteria. In recent years, the real-time quaking-induced conversion (RT-QuIC) has been included in the diagnostic criteria as a specific new biomarker criterion [12, 13]. In the presence of PrP^Sc^ seeds, RT-QuIC initiates and maintains in vitro protein misfolding of recombinant PrP by constant quaking, and facilitates a fluorescent readout. It was first established in 2011 [14] in cerebrospinal fluid and based on earlier methods using cyclic amplification [15]. The method is also able to detected PrP^Sc^ seeding activity in other tissues such as olfactory mucosa, skin, and retina [16]. The diagnostic accuracy of CSF RT-QuIC was evaluated in multiple studies that reported sensitivities between 77 and 97% and specificities of > 99% [12]. Due to its high specificity, the test facilitates early and accurate identification of sCJD. Similar to what was shown for 14-3-3 tests [17], application of RT-QuIC will most likely result in improved CJD surveillance [18, 19]. However, the method seems to be less sensitive in rare sCJD subtypes, e.g., MM2C and VV1 [19, 20]. Most important, few false-positive cases have been described [21], but the factors that may lead to such results have not been investigated in prospective studies.

Thus, we aimed to investigate the diagnostic accuracy of CSF RT-QuIC in a large-scaled prospective surveillance study over eight years and included analyses of factors that may influence the sensitivity and the specificity of the test. Further, we studied the development of sCJD incidence rates in Germany in the context of the new diagnostic protocol as well as under the impression of the COVID-19 pandemic.

## Methods

### German Creutzfeldt–Jakob disease surveillance

The German CJD Surveillance group at the University Medical Center Göttingen has systematically recorded biomarker and other clinical data of suspected prion disease patients since 1993. In 2006, it was named “National Reference Center for Transmissible Spongiform Encephalopathies” and was officially tasked with German CJD Surveillance by the Robert-Koch Institute and the German Federal Ministry of health. In Germany, suspected sporadic or acquired prion diseases are notifiable and local health care authorities contact the CJD Surveillance group for clinical case classifications. Part of these are based on personal visitation by physicians from the CJD Surveillance group and referrals to the in- and outpatient clinics of our University hospital. Especially in recent years, the majority of case classification was based on structured telephone interviews, written medical reports, and MRI image files that had been sent to the CJD Surveillance group. In addition, the majority of CSF 14-3-3 and all CSF-RT-QuIC analyses in Germany are performed in our reference laboratory. For the post-mortem case classification, the CJD surveillance group cooperates with reference centers for the neuropathology of prion diseases at the Saarland University Medical Center and at the University Medical Center Hamburg-Eppendorf.

### Study design and case classification

**Fig. 1:**
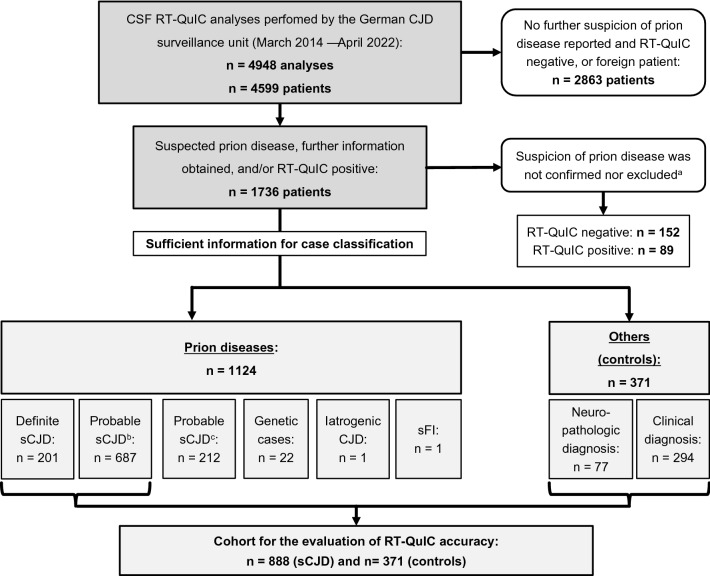
Study cohort (March 2014–April 2022). *RT-QuIC* real-time quaking-induced conversion, *sCJD* sporadic Creutzfeldt–Jakob disease, *sFI* sporadic fatal insomnia. ^a^No or insufficient clinical data for case classification available. ^b^Definite and probable sCJD according to WHO criteria [10, 11]. ^c^Probable sCJD according to amended criteria based on RT-QuIC [12], not meeting previous criteria

The presented case–control study on the diagnostic accuracy of CSF-RT-QuIC considered all diagnostic tests that were performed prospectively by the German CJD surveillance group between March 2014 and April 2022. In that timeframe, *n* = 4948 analyses were performed in 4599 patients. However, only patients with available clinical information, as well as all RT-QuIC positives (regardless of availability of further information) were considered for further investigations (*n* = 1736). In a subset of these patients (*n* = 241), the diagnosis remained unclarified due to several reasons. Patients passed before completion of clinical diagnostics and no autopsy was performed; requested case data was not sent by the treating institution; available data on diagnostic measures was not complete or not conclusive. In this study, clinically characterized patient groups were classified as follows (Fig. 1):

- Definite and probable CJD were diagnosed according consensus criteria from 2009 [11] without consideration of RT-QuIC results (*n* = 888).
- Control patients (*n* = 371) were classified as non-prion disease cases when either neuropathological investigation or clinical characteristics (including CSF analyses and neuroimaging) undoubtedly indicated other etiologies.

Patients with probable sCJD based on RT-QuIC positivity [12] not meeting pre-RT-QuIC criteria (*n* = 212) [11] were excluded from evaluations of the RT-QuIC accuracy to avoid a verification bias. Iatrogenic CJD, sporadic Familial Insomnia, and Genetic prion diseases were diagnosed based on consensus clinical or neuropathological criteria (*n* = 24) and not considered for evaluations of test accuracy as well.

Part of the data presented in this manuscript was used for previous analyses of amended diagnostic criteria and for an international validation study. This concerns *n* = 118 controls and *n* = 65 autopsy-confirmed sCJD cases from the years 2014 to 2017 [18], as well as *n* = 91 autopsy-confirmed sCJD cases and *n* = 55 controls from the years 2017 to 2019 [13].

### CSF RT-QuIC and other diagnostics

Proteins 14-3-3 were detected by Western Blot until March 2016 and measured by ELISA since April 2016, according to previously published protocols [22, 23]. EEGs and MRIs were considered for case classification according to established criteria [12] when images were reviewed by physicians from the German CJD Surveillance or when written and detailed neuroradiological reports were available.

CSF RT-QuIC was performed if requested by treating institutions or routinely as a validation test in all 14-3-3 positive samples, and when treating institutions or the surveillance group expressed clinical suspicion of prion disease. A previously published and validated test protocol using hamster-sheep chimeric recombinant PrP was applied [18, 24, 25]. The assay was rated positive when > 50% of the replicates (two of three) showed a fluorescence signal increase by more than 10,000 relative fluorescence units from the assay’s initial baseline before 80 h passed. For each replicate, 14 µl CSF was processed (45 µl in total). Since August 2018, assays with positive result in one of the three replicates were repeated according to the same protocol. If so, the overall test was rated positive, when more than two replicates were positive in six runs (≥ 50%). All technicians performed all CSF tests blind to clinical data. Neuropathological diagnosis was based on histochemical PrP^Sc^ subtyping [26] in the majority and a membrane adsorption assay as screening method [27], followed by PrP^Sc^-detection by Western Blot assay in a subset of the patients.

### Statistical methods

We calculated test sensitivity and specificity with 95% confidence intervals (95%CI) and applied Fisher’s exact test to investigate distribution of positive and negative RT-QuIC results among groups with defined characteristics. The two-tailed Mann–Whitney *U* Tests was applied to compare non-parametric data between positive and negative cases. Statistical significance was considered at *p* < 0.05. Statistics were performed with SPSS and graphics were created with Microsoft Office.

## Results

### Descriptive data and test accuracy

The study for evaluation of RT-QuIC in sCJD included data from *n* = 1259 patients (51% females) with a median age of 70 years (min: 8, max: 94) at lumbar puncture. The biggest proportion was diagnosed either as definite sCJD (*n* = 201) or probable sCJD (*n* = 687). In sCJD patients (*n* = 888, 48% females, n = 201 definite and n = 687 probable cases), the median age was 69 years (min: 43, max: 94). The data are shown in Table 1 and a graphical summary of the study cohort is presented in Fig. 1. Other groups with prion disease are shown in Supplementary Table 1. The time from onset to lumbar puncture was estimated in sCJD patients based on arrival of CSF samples at our center and reports of disease onset by caregivers or relatives. Here, the median time from onset to RT-QuIC test was 62 days (min: 2, max: 1163, n = 830). The median time from test to death was 20 days (min: 0, max: 962, *n* = 553). Out of sCJD patients with known disease duration *n* = 111 (20%) were tested in the first and n = 439 (80%) in the second half of the total disease course.

**Table 1:** Demographics and results of RT-QuIC analyses in sCJD and control patients

	*n*	Sex (f/m)	Age median (min–max)	RT-QuIC positive	RT-QuIC negative	Sensitivity (95% CI)
All cases and controls	1259	637/622	70 (8–94)	806	453	–
All sCJD	888	424/464	69 (43–94)	801	87	90.2% (88.06–92.08)
Definite sCJD	201	78/123	67 (43–86)	178	23	88.6% (83.33–92.61)
MM/MV1	94	52/42	67 (49–86)	83	11	88.3% (80.03–94.01)
MM/MV1 + 2C	23	8/15	68 (53–83)	21	2	91% (71.96–98.93)
VV2	19	8/11	66 (44–78)	19	0	100% (82.35–100)
MV2K	12	6/6	68 (43–76)	11	1	92% (61.52–99.79)
MM/MV2C	8	3/5	56 (51–67)	5	3	63% (24.49–91.48)
VV1	5	1/4	68 (57–82)	3	2	60% (14.66–94.73)
Probable sCJD	687	346/341	70 (44–94)	623	64	90.7% (88.26–92.75)

						Specificity (95% CI)
Non-CJD (all)	371	213/158	73 (8–92)	5	366	98.7% (96.9–99.56)
Non-CJD (clinical)^a^	294	173/121	73 (20–92)	4	290	98.6% (96.55–99.63)
Non-CJD (definite)^b^	77	40/37	72 (8–85)	1	76	98.7% (92.98–99.97)

^a^Clear clinical evidence for other diagnosis explaining the patient’s condition

^b^Autopsy excluding prion disease or biopsy revealing other diagnosis. sCJD: sporadic Creutzfeldt–Jakob disease; definite probable, and possible sCJD: WHO criteria [10, 11]

The sensitivity of RT-QuIC was evaluated in each group with prion disease and the specificity was calculated in non-prion disease control patients as presented in Table 1. In summary, CSF RT-QuIC showed a sensitivity of 90.2% (95% CI 88.06–92.08) for sCJD (90.7% in probable and 88.6 in definite sCJD). The sensitivity was highest in the most frequent “classical” (MM/MV1: 88%, MM/MV1 + 2C: 91%) and “ataxic” (MV2K: 92%, VV2: 100%) sCJD subtypes. In rare subtypes with predominant cortical pathology, CSF RT-QuIC showed lower sensitivities (MM/MV2: 63%, VV1: 60%). The specificity of CSF RT-QuIC was 98.7% (95% CI: 96.90–99.56) in all control cases and 98.7% (95% CI 92.98–99.97) when only autopsy-confirmed cases were considered.

### RT-QuIC sensitivity in sCJD

Regarding the rate of true-positive RT-QuIC results, we observed no significant differences between males and females (*p* = 0.911), as well as between definite and probably sCJD (*p* = 0.316). In addition, the age at sampling was not significantly different between RT-QuIC-positive and -negative sCJD patients. In contrast, we observed differences regarding total disease duration (longer disease duration in RT-QuIC-negative patients, *p* < 0.001) and disease stage (higher rate of false negatives in early clinical stages, when patients did not fulfill clinical WHO criteria [10], *p* = 0.029). Information on the sCJD subtype was available in *n* = 161 cases with neuropathology-based diagnosis. In 40 out of the 201 cases with definite diagnosis, PrP^Sc^ was detected by immune-histochemistry or Western Blot, but further characterization was not possible due to low tissue volume (biopsy), autolysis, or missing genetic information. Codon 129 testing was performed in *n* = 114 patients with definite or probable sCJD. The sensitivity was similar between M/M, M/V, and V/V (83–86%). A summary is shown in Table 2.

**Table 2:** Sensitivity of RT-QuIC in sCJD: Demographics, disease stage, and Codon 129

	*N*	Positives	Negatives	Sensitivity	*p* value
All sCJD: Age at sampling [median years (min–max)]	888	69 (43–94)	68 (48–89)	–	0.370^c^
Definite sCJD: Age at sampling [median years (min–max)]	201	67 (43–86)	65 (48–84)	–	0.218^c^
All sCJD: Total disease duration [median days (min–max)]	550	81 (18–871)	133 (32–1409)	–	< 0.001^c^
Definite sCJD: Total disease duration [median days (min–max)]	176	80 (22–871)	160 (42–1409)	–	0.006^c^
Syndrome at sampling: Early/late^a^	62/139	50/128	12/ 11	81%/92%	0.029^d^
Tested in 1st/2nd disease half^b^	36/141	29/127	7/14	81%/90%	0.146^d^
All sCJD: female/ male	888	383/418	41/46	90%/90%	0.911^d^
Definite sCJD: female/ male	201	69/109	9/14	88%/89%	1^d^
Definite sCJD/probable sCJD	201/687	178/623	23/64	89%/91%	0.418^d^
All CJD: Codon 129 M/M	60	51	9	85%	–
All CJD: Codon 129 M/V	42	36	6	86%	–
All CJD: Codon 129 V/V	12	10	2	83%	–

^a^Clinical signs according to WHO diagnostic criteria at time of lumbar puncture in later autopsy-confirmed cases. Early: Clinical WHO criteria not fulfilled; late: Probable sCJD (full syndrome with at least 3 different neurological signs); sufficient clinical data were not available in *n* = 4 confirmed sCJD cases

^b^Total disease duration was determined from onset to death and samples that were analyzed within the lower 50% (number of days) of the total duration were assigned to “tested in 1st disease half”

^c^Mann–Whtiney *U* Test (two-tailed, positives vs. negatives)

^d^Fisher’s exact test on distribution of positive and negative RT-QuIC results among groups (two-tailed)

Further, we investigated the sensitivity of biomarker combinations in an explorative manner in sCJD cases with neuropathological confirmation. We observed high sensitivities for CSF RT-QuIC (89%), 14-3-3 (89%), and MRI (83%), whereas EEG showed a weak sensitivity (40%) and was not considered for further evaluations. Combining two or more biomarkers increased the sensitivity to 95% (RT-QuIC, 14-3-3), 99% (RT-QuIC, MRI), and 100% (RT-QuIC, MRI, 14-3-3), respectively (Table 3).

**Table 3:** Sensitivity of biomarkers and combinations in definite sCJD

Biomarker/combination	*N*	Positive	Negative	Sensitivity (%)
CSF RT-QuIC	201	178	23	89
CSF 14-3-3	200	178	22	89
MRI	182	151	31	83
MRI^a^	129	119	10	92
MRI^b^	53	32	21	60
EEG	184	74	110	40
RT-QuIC or 14-3-3 positive	200	190	10	95
RT-QuIC or MRI positive	182	181	1	99
RT-QuIC or 14-3-3 or MRI positive	182	182	0	100

^a^Only MRI scans that were reviewed by the CJD Surveillance group

^b^MRI scans that were solely reviewed by local radiologists; two-tailed Fisher’s exact test: MRI positives vs. negatives (categories) in MRI reviewed in the CJD Surveillance group vs. external MRI reports (groups): *p* < 0.001

Additionally, we observed was a significantly different rate (*p* < 0.001) of true-positive MRI results between image evaluations by the CJD Surveillance group (92%) and external physicians (60%), leading to the combined sensitivity of 83%. Ten scans that were rated negative by the surveillance group showed either no restricted diffusion, ambiguous presence of restricted diffusion or cortical ribboning in only one region, which did not fulfill the applied criteria [12]. External false negative (written) reports indicated either no abnormalities (most commonly) or interpreted restricted diffusion or FLAIR hyperintensities as signs of ischemia or encephalitis. Surveillance group evaluations used images from local scans or DICOM data files from external sources. These evaluations were performed blind to external reports and final diagnosis but in the framework of the ongoing diagnostic procedure. Thus, this observation is not a result of a controlled and specific study, but a finding that is in line with a recent report from the UK [28].

### RT-QuIC in non-prion disease control patients

**Fig. 2:**
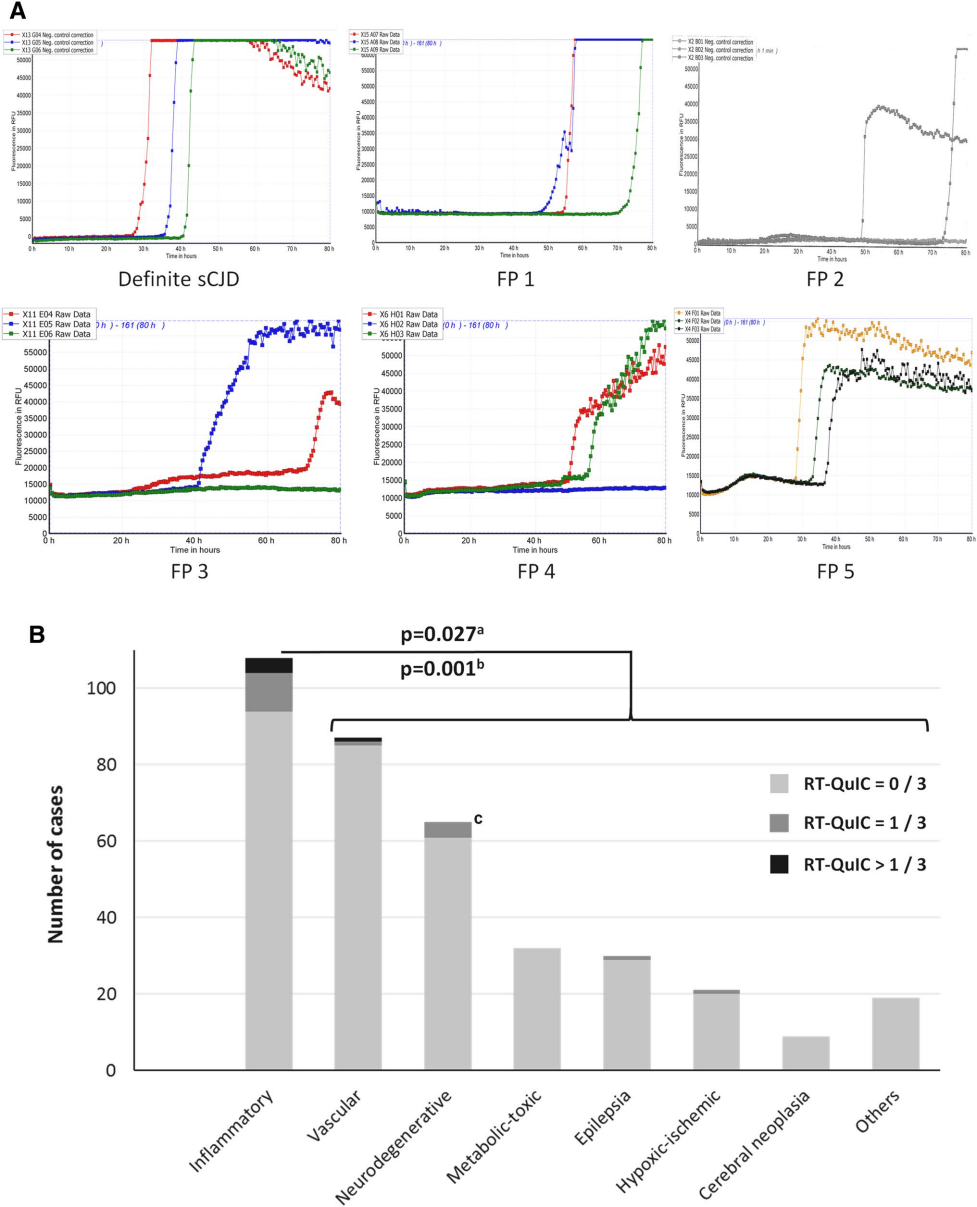
False-positive CSF RT-QuIC fluorescence signals in control patients. **A** The images show fluorescence signal (relative fluorescence units, y-axis) development over time (until 80 h after test initiation, *x*-axis). According to our test protocol, each patient was analyzed in triplets. An example of a typical RT-QuIC reaction is shown in the upper left corner. The false-positive sample from a patient with brain sinus thrombosis is shown in the upper right corner (FP 1). Four patients with encephalitis are shown below (FP2–FP5). **B** The bar chart includes categories of differential (non-prion) diagnoses. Light gray: Cases without positive signal. Dark gray: Positive RT-QuIC signal in one of the three replicates. Black: Positive signal in at least two of the three replicates. ^a^Fisher’s exact test on distribution of positive (> 1/3) and negative (≤ 1/3) RT-QuIC tests among patients with and without inflammatory brain diseases. ^b^Fisher’s exact test on distribution of at least partial (> 0/3) and no (0/3) detection of PrP^Sc^ seeding activity reactions among patients with and without inflammatory brain diseases. ^c^One patient with false-positive RT-QuIC and inflammatory CSF and transient response to immune-therapy had also evidence for a (FUS-positive) neurodegenerative disease in biopsy

The specificity of CSF RT-QuIC was investigated in *n* = 371 control patients, of which five (1.3%) were false positive. Within the subgroup of controls with neuropathological diagnosis (*n* = 77), only one patient showed false-positive RT-QuIC. The patient had an inflammatory CSF syndrome and brain biopsy revealed meningeal and perivascular lymphocytic infiltrations but also cellular FUS-positive inclusion bodies. In the end, the relationship between neuro-inflammation, neurodegenerative pathology, and clinical phenotype could not be clarified. Out of four false-positive patients with clinical diagnosis, one had a brain sinus thrombosis and the others were diagnosed with immune mediated or unclear encephalitis. In other words, the specificity of CSF RT-QuIC was 96.3% when inflammatory CNS diseases and 99.6% when other diagnoses were used as controls, respectively. All of the four encephalitis patients showed at least partial clinical improvement after steroid treatment or plasmapheresis. Regarding results from basic CSF analyses, some patients showed signs of inflammation such as slightly elevated white blood cell count or oligoclonal bands and some did not. None of the false-positive cases showed CJD-typical findings on MRI. In some cases, repetitive testing from the same sample showed consistent (false-positive) reaction (Table 4). The fluorescence signal curves in false-positive cases showed some abnormalities compared to sCJD. Patients with encephalitis displayed fluorescent curves with either fluctuating baseline signal, rather flat evolution of signal increase, or lower signal maximum than usual, although a significant increase above the pre-defined cut-off was present. In contrast, the patient with brain sinus thrombosis resembled a CJD-typical RT-QuIC reaction but rather late signal increase (Fig. 2A).

**Table 4:** Biomarker and clinical characteristics of patients with false-positive RT-QuIC

	FP 1	FP2	FP 3	FP 4	FP 5
Diagnosis	Brain sinus thrombosis	Paraneoplastic encephalopathy^a^ (Ovarian carcinoma)	Autoimmune encephalitis (unknown cause)	Autoimmune encephalitis/FTD-FUS^b^	Autoimmune encephalitis (SREAT)
Initial symptoms	Seizures, memory deficits	Seizures, global encephalopathy	Cerebellar syndrome	Psychosis, speech deficits	One seizure, memory deficits
Response to steroids or plasmapheresis	n.a	Temporary clinical improvement	Permanent cease of symptom progression	Temporary clinical improvement	Permanent full regression of symptoms
MRI^c^	Negative	Negative	Negative	Negative	Negative
CSF					
1st RT-QuIC (all replicates)	Positive 3/3 (9/9)	Positive 2/3 (5/6)	Positive 2/3 (2/3)	Positive 2/3 (3/6)	Positive 3/3 (3/3)
2nd RT-QuIC	n.a	n.a	(Negative 0/3)	(Negative 0/3)	(Negative 0/3)
14-3-3^d^	Weak positive	Negative	Positive	Negative	Positive
White blood cells	9/mm^3^	“not elevated”	9/mm^3^	3/mm^3^	3/mm^3^
Oligoclonal bands	No	No	No	Yes	No
Intrathecal IgG syntheses	No	No	No	Yes	No
Specific antibodies	Not tested	None detected	None detected	anti-CV2 (weak reaction in immunoblot)	High level of TPO antibodies

*FTD* Fronto-temporal dementia, *FUS* fused in sarcoma, RNA-binding protein fused in sarcoma, *SREAT* Steroid-responsive encephalopathy associated with autoimmune thyroiditis, *LP* lumbar puncture, *DWI* diffusion weighted images, *CV2 TPO* Thyroid peroxidase

^a^Paraneoplastic disease were diagnosed based on presence of a newly diagnosed ovarian carcinoma, signs of limbic encephalitis on MRI, and clinical response to steroids. However, no specific antibody was detected

^b^One patient (FP4) had ambiguous diagnostic results from clinical and neuropathological diagnostics

^c^Magnetic Resonance Imaging: Rated “negative” if not meeting criteria for an CJD-typical MRI^11^

^d^14-3-3 and RT-QuIC tests were performed in samples from the same lumbar puncture

The rate of false-positive results was significantly increased in patients with diagnosis of inflammatory brain disease (4 of 108) compared to those without diagnosis of inflammatory CNS diseases (1 of 263, Fisher’s exact test: *p* = 0.027, Fig. 2B). Thus, we further investigated the general frequency of RT-QuIC signal increase among controls. The assay has been performed in triplicates in our center as described above and was only rated positive, when at least two of these replicates showed an increased signal above the threshold. On the other hand, many of the sCJD patients with negative tests results (47%) and several control patients (5%) showed a signal increase in only one of the replicates. We analyzed the occurrence of these signal increases in control patients. The biggest diagnostic group included inflammatory CNS diseases (*n* = 108), followed by neurovascular (*n* = 87) and neurodegenerative diseases (*n* = 65, *n* = 66 when one patient with concurring inflammatory and neurodegenerative etiology was also assigned to this group). The overall rate of false-positive results and single RT-QuIC signal increases was significantly higher in patients with inflammatory CNS diseases than in those without (Fisher’s exact test: *p* = 0.001, Fig. 2B, Supplementary Table 2). Sex (Fisher’s exact test: *p* = 0.510) and age (Mann–Whitney U test: p = 0.473) did not differ significantly between the non-prion disease groups with all negative RT-QuIC replicates (*n* = 349) and at least one positive RT-QuIC replicate (*n* = 22).

### Serial RT-QuIC tests

Subsequent lumbar punctures and diagnostic tests we performed in a subset of patients under the same analytic conditions and similar sample characteristics (no relevant blood contamination). Most of these tests were either repeatedly negative (*n* = 68) or positive (*n* = 28). In *n* = 33 cases with sCJD and one with GSS, the initial RT-QuIC tests were negative but later tests were positive. Interestingly, RT-QuIC turned negative in the disease course of *n* = 5 cases (Table 5). Three were (initially false-positive) control patients with encephalitis, which turned RT-QuIC negative after steroid or plasmapheresis treatment. Two other cases were diagnosed with probable sCJD. In these two patients, all other clinical markers (syndrome, EEG, CSF 14-3-3, and MRI) showed CJD-typical characteristics. We could not identify analytical reasons for the discrepancy but pre-analytical differences between the samples (before arrival at our center) or stochastic effects may be potential explanations.

**Table 5:** Diagnoses of patients with false-positive CSF Quaking-Induced Conversion

	Controls (*n*)	False positives	Diagnoses
McGuire et al. 2012 [32]	103	1	Clinical diagnosis of vascular dementia
Cramm et al. 2016 [25]	400	2	Clinical diagnosis of AD Unclarified diagnosis (prion disease not excluded)
Lattanzio et al. 2017 [35]	348	2	Clinical diagnosis of paraneoplastic encephalopathy Clinical diagnosis of frontotemporal dementia
Foutz et al. 2017 [34]	67	1	Autopsy-confirmed DLB (but also low PrP^Sc^ levels in Western Blot)
Hayashi et al. 2016 [37]	n.a	1	Autopsy-confirmed FTLD-TDP type A with upper motor neuron disease
Budhram et al. 2019 [39]	14	1	Autopsy-confirmed amyloid beta-related angiitis (Endpoint Quaking-Induced Conversion)
Hayashi et al. 2017 [21]	n.a	1	Steroid-responsive encephalopathy
Rhoads et al. 2020 [19]	69	1	Autopsy-confirmed mixed (Alzheimer and cerebrovascular) disease
Simon et al. 2021 [38]	501	5	2 patients with diagnosis of autoimmune encephalopathy 3 patients with unknown diagnosis (prion disease not suspected) (Endpoint Quaking-Induced Conversion)

### Development of CJD incidence in Germany (2006–2021)

**Fig. 3:**
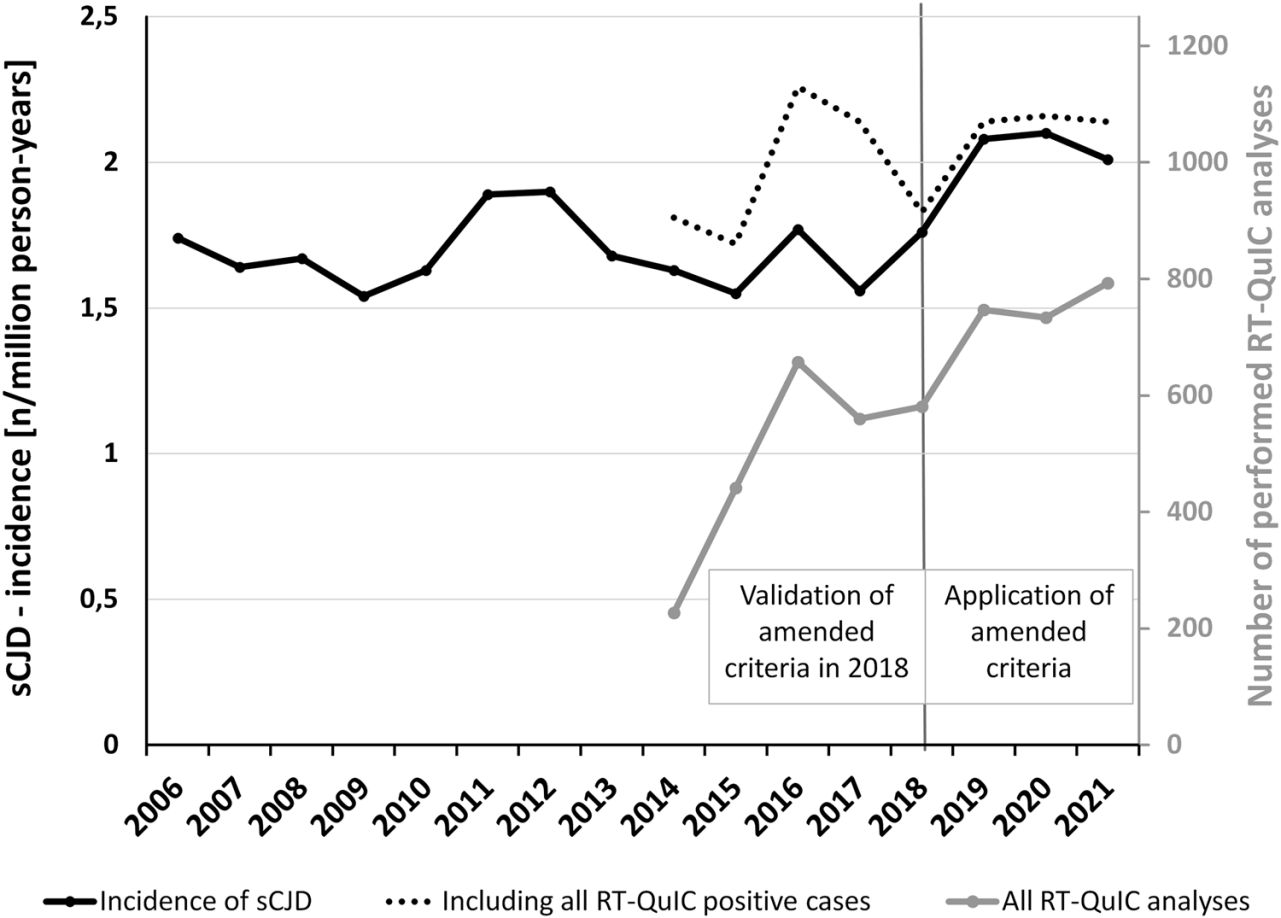
Annual incidence of sporadic CJD in Germany (2006–2021). The figure displays the development of the annual German sCJD-Incidence (black line) and the according incidence when all RT-QuIC-positive patients were considered (black dots) in cases per million person-years (left *y*-axis). Before 2018, the discrepancy is mostly due to application of pre-RT-QuIC criteria. Other reasons were positive RT-QuIC tests without available clinical data for case classification (in total *n* = 84 since 2014) and very few false-positive patients (*n* = 5 since 2014). In addition, the figure indicates the number of all performed CSF RT-QuIC analyses (gray line, right *y*-axis)

The cumulative incidence of sCJD has increased from 1.7 per million person-years 2006–2017 to 2.0 per million person-years in 2018–2021, when diagnostic criteria including RT-QuIC were applied prospectively (Fig. 3). These data also include sCJD cases that were classified without RT-QuIC analyses (based on 14-3-3, MRI, and EEG only) as well as autopsy results from cases without available clinical data. In some RT-QuIC-positive cases, no further or no sufficient clinical information was available to the CJD Surveillance group. These cases were indicated as “unclarified” throughout this manuscript. Including these patients resulted in a cumulative incidence of 2.1 per million person-years (2018–2021). We could not observe any suggestive alteration of sCJD incidence in the years of the COVID-19 pandemic 2020 (2.10) and 2021 (2.01) compared to the preceding year 2019 (2.08), the first year in which all cases were systematically classified according to the amended criteria.

## Discussion

CSF RT-QuIC has become the gold standard in the laboratory-based diagnosis of sCJD [29]. It is currently applied as a solitary criterion within the biomarker-set of diagnostic protocols [12] and as a “confirmatory” test after CJD-typical results from other biomarker analyses [30]. Here, we present comprehensive data from a well-established surveillance system including all patients with diagnosis of sCJD (*n* = 888) and a highly specific cohort of CJD mimics (*n* = 371).

We observed a sensitivity of 90% for sporadic sCJD, which is comparable to some reports from surveillance centers using 1st generation RT-QuIC [31, 32] and slightly below the sensitivity of a modified protocol called 2nd generation RT-QuIC (IQ), which was introduced in 2015 [33] and showed a sensitivity ranging from 92 to 96% [33–35]. Several factors that may influence the test sensitivity have been proposed in the past. We observed no significant differences between true and false-positive sCJD patients regarding age and sex, in contrast to a previous study that identified false-negative results to be associated with lower age and female sex [19]. We can only speculate on the reasons for this discrepancy, but it could possibly be associated with different test protocols or with the investigated sCJD cohorts. In that study, only autopsy-confirmed cases were evaluated and we observed differences of the age and the sex distribution between probable and definite cases in our cohort (Table 2). We observed no difference of the overall test sensitivity between probable and definite patients in our cohort, though. However, we could replicate an association between longer disease duration and test negativity, and showed that false-negative RT-QuIC is associated with early disease stage. We also validated high sensitivity for the most frequent MM/MV1, VV2, and MV2 sCJD subtypes [19, 34, 35], whereas sensitivity seems to be lower in the rare MM2 and VV1 subtypes [19, 34]. In some studies [34], sensitivity was slightly higher than in ours, possibly due to different substrates or test protocols. On the other hand, investigated case numbers of these subtypes were rather low in all studies (around 10 or less in each), which may not allow to draw final conclusions. An explanation for the low sensitivity may be that MM2C and VV1 subtypes show predominant cortical PrP^Sc^ pathology in early disease stages [6] and slower disease progression than most other sCJD subtypes, possibly resulting in less amount of PrP^Sc^ in the CSF. This would be in line with evaluations in genetic prion diseases that reported low sensitivity in entities with slow disease progression (GSS) or pathology restricted to defined structures (brainstem and thalamus in FFI, cerebellum in GSS) in early disease stages [36].

Regarding the specificity of RT-QuIC, previous analyses showed an excellent accuracy of CSF RT-QuIC of about 99% or higher [12]. Our data from eight years of clinical application indicated a specificity of 99% but for the first time, we evaluated test-related and clinical data in a series of five false-positive cases. Four of them were diagnosed with immune-mediated encephalitis and the rate of false positives was significantly higher than in other diagnostic groups (*p* = 0.027). In addition, single (one of the three) false-positive signal increases were also significantly more frequent among differential diagnoses with inflammatory pathophysiologic background (*p* = 0.001), suggesting a potential causal relationship between encephalitis and false-positive RT-QuIC results. Of course, analytical and pre-analytical factors cannot be excluded. In the literature, only 10 cases of false-positive RT-QuIC or Endpoint-QuIC with clear diagnosis have been reported (see Table 5). They were diagnosed with vascular dementia [32], Alzheimer’s disease [25], mixed dementia [19], and tauopathies [35, 37], immune-mediated encephalitis [20, 35, 38], and amyloid-associated vasculitis [39].

The possibility of a higher likelihood of false-positive RT-QuIC among encephalitis patients is an important issue because many inflammatory encephalopathies are highly treatable and may represent the most important clinical mimics of CJD and causes of rapidly progressive dementia [40]. On the other hand, all false-positive patients showed either clinical or CSF characteristics that pointed to other diagnoses than CJD, indicating that consideration of factors such as inflammatory signs in the CSF may improve the specificity of an RT-QuIC-based clinical diagnosis. Total CSF Tau protein may also give additional clues because of better specificity in the discrimination of CJD and acute encephalopathies than 14-3-3, but Tau was not available for the false-positive patients. On the other hand, total-tau may also be extremely elevated in encephalitis [41] due to ongoing severe neuronal damage. More important, none of the patients showed CJD-typical MRI. In our autopsy series, no patient received incorrect ante-mortem diagnosis of CJD based on RT-QuIC positivity. We identified only five false-positive results in eight years of RT-QuIC application in a sum of 4599 patients. However, clinical information was only available for 371 control patients, leading to the reported specificity of 99%.

Further investigations have to validate our findings about false-positive RT-QuIC and investigate potential mechanisms. So far, previous studies have not found association of RT-QuIC efficiency and neuronal damage markers such as total-tau and proteins 14-3-3 in sCJD patients [42]. Total PrP was also not associated with seeding efficiency in sCJD [42] but has not been investigated as a factor for false-positive RT-QuIC, yet. On the other hand, total PrP was not shown to be significantly altered in encephalitis compared to cerebral ischemia or control patients [43]. Another potential reason may be the influence of factors in the CSF that are directly linked to neuro-inflammation. Epileptic activity in encephalitis patients was also discussed as a cause for false-positive results [21]. Our data did not allow the evaluation of the presence of seizures in relation to lumbar puncture in control patients, but patients with primary diagnosis of seizures or status epilepticus caused by idiopathic epilepsy syndromes, or reversible conditions such as alcohol withdrawal showed a low frequency of positive test replicates (one in 30 patients). However, the mechanisms for false-positive results may be related to the CSF. RT-QuIC from other body tissues such as olfactory mucosa [44, 45] are an alternative clinical test and should be investigated in future studies on the specificity of RT-QuIC in neuro-inflammatory diseases. Clarification of the reasons for false-positive PrP^Sc^ RT-QuIC reactions may also be highly relevant for the application of other protein amplification assays such as α-Synuclein RT-QuIC. So far, only very few false-positive results have been reported and were associated with Wernicke’s encephalopathy, Alzheimer’s disease, and encephalitis [46, 47].

Although our study provides comprehensive data on clinical experience with RT-QuIC, the study has naturally some limitations. First, our test protocol [24] uses chimeric hamster-sheep recombinant PrP as substrate, whereas most other centers are using hamster recombinant PrP [32, 33]. Some centers showed that IQ-CSF RT-QuIC may have a superior sensitivity for sCJD compared to previous protocols [30, 45, 48]. It remains unclear, whether IQ RT-QuIC underlays the same or similar confounders for PrP seeding as our protocol but international ring trials have shown that RT-QuIC results are highly concordant among different test centers and test protocols [25, 49]. Our protocol has been well established over years and we have achieved a high level of experience to perform the test in a reliable and reproducible way.

Regarding the sensitivity, further histochemical characterization of sCJD was only available in a limited number of cases (*n* = 161), discouraging reliable conclusions on the sensitivity of rare (MM2, VV1) sCJD subtypes. Similarly, Codon 129 was analyzed in a rather small subset of patients (*n* = 114). Lastly, our surveillance data includes a number of uncharacterized cases with suspected prion disease or positive CSF RT-QuIC. In these cases, further clinical data were not available and thus, we cannot exclude additional false-positive or false-negative test results in this group. Our cohort may be prone to an according selection bias. On the other hand, we assume that this bias might rather be less relevant than in retrospective case–control studies or in studies including only autopsy-confirmed cases.

As a secondary outcome, we observed an increased overall sCJD incidence in Germany after inclusion of the test in the clinical diagnostic protocol. In this context, we assume that the increase from 1.7 (2009–2017) to 2.0 (2018–2021) person-years is a result of the improved clinical detection of early sCJD cases (based on positive RT-QuIC) as previously suggested or observed by our group [18] and others [13, 17, 19]. This effect was also described in the context of previous criteria modifications [50]. Another interesting observation was the apparent lack of an alteration of the overall sCJD incidence during the Covid-19 pandemic. Annual numbers of positive RT-QuIC results remained stable between 2019 and 2021 (Fig. 3). Although we cannot exclude influence of viral infections on genesis or course of prion diseases, our surveillance data do not suggest an immediate causal relationship between COVID-19-related factors and CJD on the population level.

## Conclusion

Chimeric PrP CSF RT-QuIC is an accurate diagnostic tool for the differential diagnosis of sCJD. If the test is interpreted in the context of a complete diagnostic work-up, it may provide an extremely high level of ante-mortem diagnostic certainty. RT-QuIC negativity combined with absence of CJD-typical results in 14-3-3 analysis and MRI indicates extremely low likelihood of sCJD. The routine application of RT-QuIC improves CJD surveillance and leads to a formal increase of the disease incidence. However, the sensitivity is influenced by disease stage and disease subtype. False-positive results may occur and clinicians have to be aware of this possibility. In cases with ambiguous clinical presentation, we recommend consideration of other diagnostics, in particular MRI, and repetitive RT-QuIC analyses from the same sample to exclude the influence of analytical factors. Nonetheless, a consecutive lumbar puncture, at best after therapeutic intervention, may be necessary to detect false positivity based on pre-analytical or disease-related factors. In this context, RT-QuIC from other body tissues such as olfactory mucosa may be an alternative, if available. Inflammatory CNS disease, especially immune-mediated encephalitides, should always be considered as potential clinical and laboratory mimics of CJD. Although general comparability of different RT-QuIC protocols and substrates have been shown, our pilot findings need to be verified through studies using other body tissues and test protocols such as IQ.

## Supplementary Information

Below is the link to the electronic supplementary material.

Supplementary file1 (PDF 396 KB)

## Data Availability

All manuscript-related data is available and will be provided upon reasonable request.
