# Supplementary material for: Application of real-time quaking-induced conversion in Creutzfeldt–Jakob disease surveillance
Source: J Neurol. 2023 Jan 10;270(4):2149–61. doi: 10.1007/s00415-022-11549-2 (PMC9829526; doi:10.1007/s00415-022-11549-2)
Supplement: Supplementary file 1 — Supplementary file1 (PDF 396 KB) [file 415_2022_11549_MOESM1_ESM.pdf]

Supplementary Infromation

Supplementary Table 1. Demographics and results of RT-QuIC analyses in other diagnostic groups.

	n	Sex n (f/m)	Age median (min-max)	RT-QuIC positive n	RT-QuIC negative n	Sensitivity (95% CI)
Probable sCJD (RT-QuIC)[a]	212	105/107	70 (44-93)	212	0	-
Sporadic Fatal Insomnia	1	1/0	41	0	1	0%
Iatrogenic CJD	1	0/1	75	1	0	100%
Genetic prion disease	22	15/7	58 (39-89)	15	7	68.2% (45.13-86.14)
E200K (gCJD)	12	9/3	63 (52-89)	12	0	100% (73.54-100)
D178N-V (gCJD)	1	1/0	61	1	0	100%
P102L (GSS)	3	2/1	46, 58, 67	2	1	66.7%
D178N-M (FFI)	3	0/3	49, 53, 59	0	3	0%
P105L (GSS)	1	1/0	58	0	1	0%
G114V (gCJD)	1	1/0	59	0	1	0%
T183A (atypical prion disease)	1	1/0	39	0	1	0%

[a]Clinical diagnosis based on RT-QuIC positivity according to amended criteria[1,2] without fulfilling previous criteria[3] because of an incomplete clinical syndrome (not considered for evaluation of the test accuracy).
gCJD: Genetic Creutzfeldt-Jakob Diseasse; GSS: Gerstmann-Sträussler-Scheinker syndrome; FFI: Fatal Familial Insomnia

1. Hermann P, Appleby B, Brandel JP, et al. Biomarkers and diagnostic guidelines for sporadic Creutzfeldt-Jakob disease. Lancet Neurol. 2021;20:235-246. doi:10.1016/S1474-4422(20)30477-4
2. Watson N, Hermann P, Ladogana A, et al. Validation of Revised International Creutzfeldt-Jakob Disease Surveillance Network Diagnostic Criteria for Sporadic Creutzfeldt-Jakob Disease. JAMA Netw Open. 2022;5:e2146319. doi:10.1001/jamanetworkopen.2021.46319
3. Zerr I, Kallenberg K, Summers DM, et al. Updated clinical diagnostic criteria for sporadic Creutzfeldt-Jakob disease. Brain. 2009;132:2659-2668. doi:10.1093/brain/awp19

Supplementary Table 2. Non-CJD cases: Diagnoses and RT-QuIC results

Diagnosis	n	RT-QuIC: > 0 / 3[a]	RT-QuIC: (≥2/3)
**inflammatory CNS disease**	**108[b]**	**14 (13.0%)**	**4**
Encephalitis (immune-mediated)	39	4	2
Encephalitis (viral)	22	2	0
Encephalitis (unclarified cause)	32	5	2
Septic encephalopathy	11	2	0
Cerebral vasculitis	4	1	0
**vascular, non-inflammatory**	**87**	**2 (2.3%)**	**1**
Cerebral ischemia	53	0	0
VD	18	0	0
PRES	6	0	0
Intracranial hemorrhage	4	0	0
CAA	3	1	0
Brain sinus thrombosis	1	1	1
Dural arterio-venous fistula	1	0	0
Transient global amnesia	1	0	0
**neurodegenerative**	**65 (66)[b]**	**4 (6.2%)**	**0 (1)[b]**
AD	30	1	0
AD + CAA	9	1	0
Synucleinopathies (DLB, MSA)	9	1	0
Tauopathies (PSP, FTLD)	5	1	0
Spinocerebral ataxia	1	0	0
Mixed (AD + DLB)	1	0	0
Mixed (AD + VD)	10	0	0
**metabolic-toxic**	**32**	**0**	**0**
Alcohol-related (Wernicke, Korsakov)	15	0	0
Others	17	0	0
**others**	**79**	**2 (2.5%)**	**0**
Epilepsy (childhood, alcohol withdrawal, others)	30	1	0
Global hypoxic-ischemic brain damage	21	1	0
Cerebral neoplasia (glioma, lymphoma, metastatic)	9	0	0
Mitochondrial disorder	6	0	0
Psychiatric	3	0	0
Others (storage disease, hydrocephalus, GBS, concurring non-prion disease diagnoses)	10	0	0

[a]positive reactions (0-3) in RT-QuIC repetitions (percentage among all cases); [b]one patient with false positive RT-QuIC and inflammatory CSF and transient response to immune-therapy had also evidence for a (FUS-positive) neurodegenerative disease in biopsy. GBD: Guillian-Barré syndrome; VD: vascular dementia; PRES: Posterior reversible encephalopathy syndrome; AD: Alzheimer's disease; CAA: cerebral amyloid angiopathy; DLB: dementia with Lewy bodies; MSA: multi-system atrophy; PSP: progressive supranuclear palsy; bvFTLD: behavioral variant fronto-temporal lobar degeneration
